# Analysis of the variability of nursing care by pathology in a sample of nine Belgian hospitals

**DOI:** 10.1186/1472-6963-11-S1-A11

**Published:** 2011-10-19

**Authors:** M Pirson, C Delo, L Di Pierdomenico, V Biloque, D Martins, U Eryuruk, P Leclercq

**Affiliations:** 1 Health Economics, Universite Libre de Bruxelles, Ixelles, Brussels 1070, Belgium

## Introduction

In 2010, a Belgian study [1] explored the feasibility of introducing all-inclusive case-based payments for Belgian hospitals. In this kind of financing system, hospital services and patient mix are described in a simplified way through Diagnosis Related Groups (DRGs). A tariff is fixed in advance for each DRG. DRGs are groups of patients based on economic and clinical homogeneity. Clinical homogeneity is achieved on the basis of medical diagnosis, co-morbidities, medical procedures, complications, etc. Economic homogeneity is achieved by using, first of all, the length of stay (LOS) or cost (or charges) of hospitalization as a classification criterion.

As soon as DRGs were introduced, most nursing research revealed that DRGs were not very amenable to homogeneous integration with nursing care. DRGs only explained 20% to 40% of the variability in nursing care. Coefficients of variation for nursing care per DRG have been reported as varying from 0,22 to 2,56 [2-5]. This is the reason why some researchers try to refine DRG classification into classes of nursing cost per DRG [6]. However, it is difficult to find recent data that deals with this.

The objectives of this study are to:

• Discover if nursing activity is homogeneous by DRG and severity of illness.

• Evaluate the correlation between LOS of patients and nursing activity per patient.

## Methods

Nursing minimum datasets of nine hospitals were used for the year 2008. APR-DRGs of inpatients were also transmitted by hospitals. The sample is composed of 12734 complete stays (the nursing minimum dataset is only obligatory 4 * 15 days a year per hospital). The transformation of nurse activity into nursing time was made by using existing time-by-nurse statistics from two reports published by the Ministry of Public Health (Win [7] and Welame [8] reports).

To evaluate the homogeneity of nursing activity by DRG, an analysis of percentiles and coefficients-of-variation was carried out on DRGs and the severities of illness that included more than 100 patients (3135 patients). A selection of high and low outliers was also done. The 75th percentile +1,5* interquartile range was used to select high outliers; the 25th percentile -1,5* interquartile range was used to select low outliers. The Pearson coefficient was used to evaluate the correlation between nursing activity and the LOS of patients.

## Results

The heterogeneity of the nursing activity is high within DRGs. Coefficients of variation vary between 0,47 and 1,40 according to DRG. Interquartile ranges vary from 71 to 455 minutes according to DRG. The correlation between nurse activity and LOS is good (r=0,69, p<0,001). The intensity of the correlation is, however, variable from one DRG to another, varying from 0,05 (P>0,05) to 0,65 (p<0,001). The percentage of LOS outliers is more important than the percentage of nursing activity outliers (5,6 against 5,2%). Only 31,10% of high nursing activity outliers are also high LOS outliers. Only 32, 48% of high LOS outliers are also high nursing activity outliers.

## Conclusions

As was foreseeable, nursing activity was proven to be heterogeneous within DRGs. This is the reason why nurses often reject all-in financing systems. Nevertheless, the variability of LOS inside DRGs seems quite as important, and LOS by DRG is, however, the basis of the funding system in Belgium (justified days). Using such an argument to reject all-in systems is not very scientific. The weight of the nursing minimum dataset is marginal inside hospital budgets.

The complete study upon which this abstract is based will thoroughly analyze the variability of activity, hospital by hospital, in order to neutralize the coding effect. As well, an analysis of outliers’ profiles will be carried out.

## References

[B1] Sermeus W Gillet P Tambeur W Gillain D Grietens J Laport N Financement des soins infirmiers hospitaliers Health Services Research (HSR) 2007 Bruxelles: Centre fédéral d'expertise des soins de santé (KCE) KCE reports 53B (D/2006/10.273/07)

[B2] McKibben RC Brimmer PF Galiher JM Hartley SS Clinton J Nursing costs and DRG payments American Journal of Nursing 1985 85 12 1353 1356 3934973

[B3] Mowry MN Korpman RA Do DRG reimbursement rates reflect nursing costs? Journal of Nursing Administration 1985 15 7,8 29 35 3926966

[B4] Sovie M Tarcinale M Vanputte A Stunden A Amalgam of nursing acuity, DRG's and costs Nursing Management 1985 16 3 22 42 3919345

[B5] Wolf GA Lesic LK Determining the cost of nursing care within DRGs. In: Shaffer FA. Eds.Patients & purse strings: patient classification and cost management 1986 New York: National League for Nursing 165 180

[B6] Sermeus W Gillet P Tambeur W Gillain D Grietens J Laport N Financement des soins infirmiers hospitaliers Health Services Research (HSR) 2007 Bruxelles: Centre fédéral d'expertise des soins de santé (KCE) KCE reports 53B (D/2006/10.273/07)

[B7] Schouppe L Defloor T Gobert M Van Goubergen D Workload Indicator for Nursing Rapport SFP-FOD 2007 Brussel: Federaal Overheidsdienst Wetenschapsbeleid ten behoove van de Federale Overheidsdienst Volksgezondheid, Leefmilieu en Veiligheid van de voedselketen

[B8] Myny D Defloor T Alvarez-Irusta L Annys D Demeyere F DeVreese I Proenca V Vandermolen M Vanderwee K Van Hecke A Gobert M Rapport final Welame, FOD Wetenschapsbeleid, Gent 282

